# Optic Neuropathy with Features Suggestive of Optic Neuritis in Cerebrotendinous Xanthomatosis

**DOI:** 10.1155/2019/2576826

**Published:** 2019-02-12

**Authors:** Miyuki Miyamoto, Nobuyuki Ishii, Hitoshi Mochizuki, Kazutaka Shiomi, Tomoko Kaida, Hideki Chuman, Masamitsu Nakazato

**Affiliations:** ^1^Division of Neurology, Respirology, Endocrinology and Metabolism, Department of Internal Medicine, University of Miyazaki, Miyazaki, Japan; ^2^Miyata Eye Hospital, Miyakonojo, Miyazaki, Japan; ^3^Department of Ophthalmology, University of Miyazaki, Miyazaki, Japan

## Abstract

We describe our encounter with a 39-year-old man who exhibited acute painless visual loss and progressive gait disturbance. He had tendinous xanthoma and several neuroophthalmological findings indicative of optic neuropathy in the right eye, including afferent pupillary defect, cecocentral scotoma, and optic disc swelling. Neurological examination showed cerebellar ataxia and pyramidal weakness. Brain magnetic resonance imaging revealed bilateral swelling in the optic nerves with gadolinium-enhancement suggesting optic neuritis, an enlarged fourth ventricle, atrophy of the cerebellum, and hyperintensities in the bilateral dentate nuclei. The patient was diagnosed with cerebrotendinous xanthomatosis (CTX) based on an elevated serum cholestanol level and a homozygous missense mutation in* CYP27A1*. CTX is a genetic lipid storage disease caused by dysfunction of the mitochondrial enzyme sterol 27-hydroxylase. With respect to ophthalmological manifestations, juvenile cataracts and optic neuropathy are common findings in patients with CTX, but there have been no reports of optic neuropathy with features suggestive of optic neuritis. Thus, this case illustrates that clinicians should consider a diagnosis of CTX in patients with cardinal features of CTX even if the patients show signs indicative of optic neuritis.

## 1. Introduction

Cerebrotendinous xanthomatosis (CTX), an autosomal recessive lipid storage disease, is characterized by various systemic and neurological symptoms, including chronic diarrhea, juvenile cataracts, tendon xanthomas, and developmental delay [1]. With respect to ophthalmological manifestations, apart from cataracts, optic neuropathy with optic disc paleness is a common finding in patients with CTX [2]. However, cases of CTX with optic neuritis have not been previously reported. Here, we present the first case of CTX with optic neuropathy and findings suggestive of optic neuritis, including disc edema and contrast agent enhancement of the peripapillary retina and optic nerve.

## 2. Case Presentation

A 39-year-old man, born of a consanguineous marriage, had epilepsy since the age of 12 years, as well as dyslipidemia; he was referred to our hospital because of acute painless visual loss and progressive gait disturbance. His visual acuity was 20/16 bilaterally after bilateral cataract surgery at the age of 31 years. His developmental milestones were normal. He denied a history of neonatal jaundice or infantile diarrhea, smoked half a pack of cigarettes per day, and drank socially. His family history was unremarkable. Medications included carbamazepine, phenytoin, clobazam, and bezafibrate.

**Figure 1:**
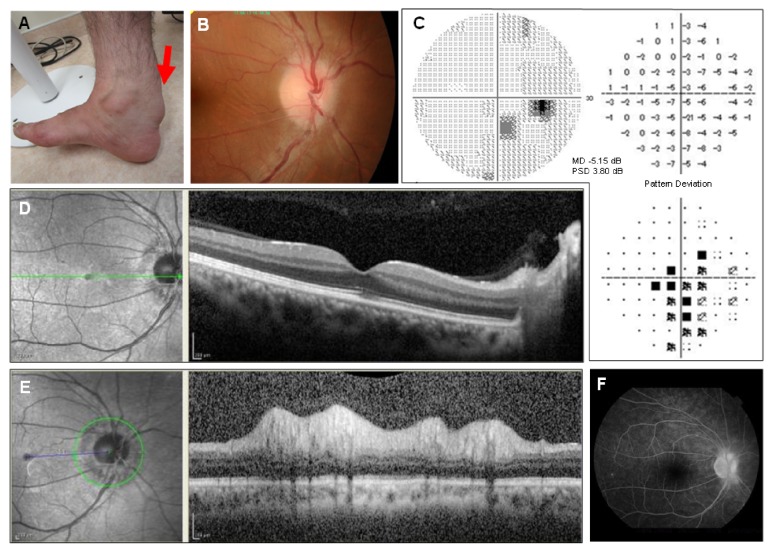
The patient's enlarged right Achilles tendon (arrow), which indicated xanthoma (A). Funduscopic examination showed mild swelling in the nasal side of the right optic disc (B). Humphrey 30-2 visual field examination revealed a cecocentral scotoma in the right eye (C). OCT of the right eye demonstrated intact thickness of the retinal nerve fiber layer (RNFL) in the macula (D) and increased thickness of the peripapillary RNFL (E). Fluorescein angiography exhibited profuse dye leakage on the right optic disc (B).

On physical examination, swelling was observed in the tendons of Achilles, patella, and triceps, which indicated xanthoma (Figure 1(A)). A neuroophthalmological examination showed corrected visual acuities of 20/125 in the right eye and 20/30 in the left eye. The right pupil measured 4 mm with a round shape in dark and 2 mm in light conditions; both pupils constricted briskly in response to light. The right eye exhibited an afferent pupillary defect. A biomicroscopic examination was remarkable for bilateral centrally-fixed intraocular lens. Funduscopic examination showed mild swelling in the nasal side of the right optic disc (Figure 1(B)); the left eye was normal. Humphrey 30-2 visual field examination showed a cecocentral scotoma in the right eye (Figure 1(C)). Optical coherence tomography (OCT) of the right eye showed intact thickness of the retinal nerve fiber layer (RNFL) in the macula (Figure 1(D)), while the peripapillary RNFL thickness had increased. Fundus fluorescein angiography (FAG) showed mild leakage on the nasal side of the right optic disc (Figure 1(F)). Pattern-reversal visual-evoked potentials (VEPs) with check sizes of 7.5', 15', 30', and 60' revealed no clear potentials in both eyes, and the P100 wave could not be identified. All findings indicated optic neuropathy. Neurological examination revealed Minimental State Examination scores of 22/30. The patient exhibited slurred speech, weakness in the lower limbs with spasticity, brisk deep tendon reflexes throughout all extremities, and bilateral positive Babinski sign. His gait was unstable because of truncal ataxia.

Routine laboratory tests revealed the following: normal renal and liver functions, no elevations in inflammatory markers such as erythrocyte sedimentation rate and C-reactive proteins, normal LDL cholesterol, 137 mg/dL, high triglycerides, 331 mg/dL, increased lactic acid levels, 17.3 mg/dL (reference range: 3.0–17.0 mg/dL), and pyruvate levels, 2.02 mg/dL (reference range: 0.3–0.94 mg/dL). Serological analyses of the herpes simplex virus, cytomegalovirus, Epstein-Barr virus, angiotensin-converting enzyme, PR3 and MPO anti-neutrophil cytoplasmic antibodies, anti-nuclear antibody, SS-A and SS-B antibodies, and anti-DNA antibody revealed negative results. Moreover, anti-aquaporin 4 and anti-myelin oligodendrocyte antibodies were not detected. No mitochondrial DNA mutations (i.e., nucleotide positions 3460, 11,778, and 14,484) associated with Leber's hereditary optic neuropathy (LHON) were observed. Cerebrospinal fluid (CSF) analysis revealed no cells but showed an increased protein level (77 mg/dL); isoelectric focusing revealed no oligoclonal bands in the CSF.

**Figure 2:**
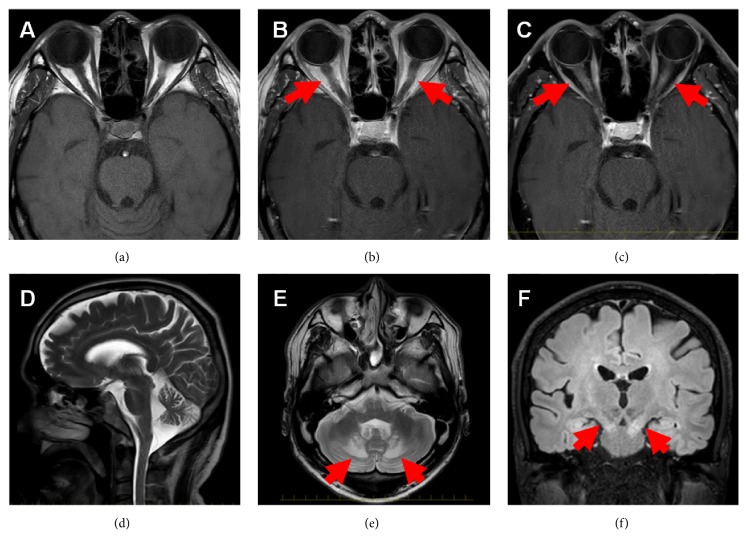
Brain magnetic resonance imaging revealed the following: bilateral swelling of optic nerves on T1-weighted images (a) with enhancement (arrows) on gadolinium-enhanced T1-weighted (b) and T1 weighted fat-suppressed images (c), enlarged fourth ventricle and cerebellar atrophy (d), and hyperintensities in the bilateral dentate nuclei (arrows, (e)) on T2-weighted images; FLAIR images demonstrated hyperintensities in the bilateral dentate nuclei and pyramidal tract (arrows, (f)).

Brain magnetic resonance imaging (MRI) revealed bilateral swelling in the optic nerves on T1-weighted images (Figure 2(a)) and enhancement on gadolinium-enhanced T1-weighted and T1-weighted fat-suppressed images (Figures 2(b) and 2(c)), suggesting optic neuritis. Furthermore, T2-weighted images showed an enlarged fourth ventricle, atrophy of the cerebellum, and hyperintensities in the bilateral dentate nuclei (Figures 2(d) and 2(e)); fluid-attenuated inversion recovery images demonstrated hyperintensities in the bilateral dentate nuclei and the pyramidal tract (Figure 2(f)).

The patient was finally diagnosed with CTX upon observance of an elevated serum cholestanol level of 13.6 *μ*g/mL (reference range: 2.0–3.4 *μ*g/mL) and a homozygous missense mutation (c.1421G>A) in* CYP27A1*.

One month later, before the initiation of oral chenodeoxycholic acid (CDCA) treatment, the patient's visual acuity spontaneously recovered to 20/16 bilaterally, and hyperintensities in the optic nerves were found to be diminished on follow-up MRI. His neurological symptoms, however, were not altered 6 months after administration of CDCA.

## 3. Discussion

CTX, an autosomal recessive lipid storage disease, is caused by mutations in* CYP27A1*, which encodes the mitochondrial enzyme sterol 27-hydroxylase [1]. A deficiency of this enzyme causes disruption of the formation of bile acid, including cholic acid and chenodeoxycholic acid. Subsequently, cholesterol and cholestanol accumulate in tissues throughout the body, leading to the development of many systemic and neurologic symptoms [1].

Ophthalmological manifestations in CTX include juvenile cataracts, the incidence of which may be up to 90% [1]. In addition, pale optic disc, exophthalmos, xanthelasma, and premature retinal senescence have been reported [1–3]. With respect to optic nerve dysfunction, optic neuropathy may occur in 50% of patients with CTX [2, 3]. Moreover, 50–80% of affected patients exhibit a prolonged or diminished VEP latency in the presence or absence of abnormal fundus [2–4]. Optic neuropathy in CTX is not uncommon; however, optic neuropathy with features suggestive of optic neuritis, including the spontaneous recovery of visual acuity and contrast enhancement of the peripapillary retina and optic nerve, as in our patient, has not been previously reported. Thus, through this case, we discuss whether this patient's optic neuropathy was caused by CTX and whether CTX is accompanied with ophthalmological findings similar to those seen in optic neuritis.

The etiology of optic neuropathy with acute visual loss includes optic neuritis, arteritic and nonarteritic ischemic optic neuropathy, infections, optic nerve compression, LHON, toxic and metabolic optic neuropathy, and traumatic optic neuropathy [5]. Considering the pathophysiology of CTX, which involves the accumulation of cholesterol and cholestanol in virtually all tissues, nonarteritic ischemic optic neuropathy (NAION) is immediately included in the differential diagnosis of a patients with optic neuropathy. However, the presence of contrast-enhanced optic nerves on MRI and spontaneous recovery of visual acuity render NAION a less likely diagnosis [6]. Except for idiopathic optic neuritis, other differential diagnoses were also not compatible with the patient's history, neuroophthalmological examination, and laboratory and imaging findings. Although we could not exclude idiopathic optic neuritis, we speculated that the patient's optic neuropathy was caused by CTX because optic neuropathy is commonly associated with CTX.

We hypothesized that optic neuropathy with findings similar to those seen in optic neuritis in CTX may involve mitochondrial dysfunction, although the exact mechanism remains unclear. Sterol 27-hydroxylase, a mitochondrial enzyme, is impaired in CTX, leading to abnormalities in mitochondrial function as well as lipid metabolism [1]. Indeed, the following findings suggesting mitochondrial dysfunction have been revealed by previous studies: increased lactic acid and pyruvate levels in the blood and CSF [7], a lactate peak on brain MR spectroscopy [8], decreased activities of mitochondrial respiratory chain enzymes [7], and structural abnormalities in the mitochondria [9].

Similarly, LHON is established as a mitochondrial disorder. A typical clinical presentation of LHON includes acute or subacute painless visual loss accompanied by disc swelling, which resembles our patient's clinical course; however, leakage in the fundus FAG and progressive optic nerve atrophy is not typical of LHON [10]. However, several studies have reported fundus edema, dye leakage in FAG, and gadolinium-enhancement of the optic nerve on MRI, masquerading as optic neuritis [11, 12], as well as spontaneous improvement in visual acuity [13] in patients with LHON. The similarities between our case and LHON cases indicate that the ophthalmological findings in our patient may have resulted from mitochondrial dysfunction in CTX. Furthermore, our patient's clinical course might explain the mechanism underlying the optic neuropathy commonly seen in CTX.

To the best of our knowledge, there have been no reports of cases of optic neuropathy with features suggestive of optic neuritis in CTX. The exact underlying mechanisms remain unclear; however, we speculate that mitochondrial dysfunction caused by CTX may be involved. Thus, this case illustrates that clinicians should consider a diagnosis of CTX in patients with cardinal features of CTX, such as xanthomas or hyperintensities of the dentate nuclei on brain MRI, even in the presence of contrast enhancement of the optic discs and optic nerves, indicating optic neuritis.
